# A system based on deep convolutional neural network improves the detection of early gastric cancer

**DOI:** 10.3389/fonc.2022.1021625

**Published:** 2022-12-22

**Authors:** Jie Feng, Shang rui Yu, Yao ping Zhang, Lina Qu, Lina Wei, Peng fei Wang, Li juan Zhu, Yanfeng Bao, Xiao gang Lei, Liang liang Gao, Yan hu Feng, Yi Yu, Xiao jun Huang

**Affiliations:** ^1^ Department of Gastroenterology, Lanzhou University Second Hospital, Lanzhou, Gansu, China; ^2^ Technology Research and Development Department, Digestive Endoscopy Engineering Research and Development Center of Gansu Province, Lanzhou, Gansu, China; ^3^ Department of Sciences and Technology, Beijing Huag gen Anbang Technology Technology Company Limited, Beijing, China; ^4^ Department of Gastroenterology, Lanzhou Cheng guan District People’s Hospital, Lanzhou, Gansu, China; ^5^ Department of Gastroenterology, Min County People’s Hospital, Ding Xi, Gansu, China

**Keywords:** deep convolutional neural network, early gastric cancer, diagnosis rate, sensitivity, accuracy, false positive, false negative

## Abstract

**Background:**

Early gastric cancer (EGC) has a high survival rate, but it is difficult to diagnosis. Recently, artificial intelligence (AI) based on deep convolutional neural network (DCNN) has made significant progress in the field of gastroenterology. The purpose of this study was to establish a DCNN assist system to improve the detection of EGC.

**Methods:**

3400 EGC and 8600 benign images were collected to train the DCNN to detect EGC. Subsequently, its diagnostic ability was compared to that of endoscopists using an independent internal test set (ITS, including 1289 images) and an external test set (ETS, including 542 images) come from three digestive center.

**Results:**

The diagnostic time of DCNN and endoscopists were 0.028s, 8.05 ± 0.21s, 7.69 ± 0.25s in ITS, and 0.028s, 7.98 ± 0.19s, 7.50 ± 0.23s in ETS, respectively. In ITS, the diagnostic sensitivity and accuracy of DCNN are 88.08%(95% confidence interval,95%CI,85.24%-90.44%), 88.60% (95%CI,86.74%-90.22%), respectively. In ETS, the diagnostic sensitivity and accuracy are 92.08% (95%CI, 87.91%- 94.94%),92.07%(95%CI, 89.46%-94.08%),respectively. DCNN outperformed all endoscopists in ETS, and had a significantly higher sensitivity than the junior endoscopists(JE)(by18.54% (95%CI, 15.64%-21.84%) in ITS, also higher than JE (by21.67%,95%CI, 16.90%-27.32%) and senior endoscopists (SE) (by2.08%, 95%CI, 0.75%-4.92%)in ETS. The accuracy of DCNN model was higher (by10.47%,95%CI, 8.91%-12.27%) than that of JE in ITS, and also higher (by14.58%,95%CI, 11.84%-17.81%; by 1.94%,95%CI,1.25%-2.96%, respectively) than JE and SE in ETS.

**Conclusion:**

The DCNN can detected more EGC images in a shorter time than the endoscopists. It will become an effective tool to assist in the detection of EGC in the near future.

## Introduction

According to the 2020 Global Cancer Statistics, Gastric cancer is the third most lethal and the fifth most common malignancy from a global perspective, and Asia remains the region with a high incidence of cancer, with a cancer incidence rate of 49.3% and a mortality rate of 58.3%, with 719524 new cases of gastric cancer (1). The survival rate of patients with stage IA was 91%, Whereas the patients with stage IV less than 17% (2) Therefore, the early detection of gastric cancer is particularly important. However, the diagnosis of early gastric cancer (EGC) is difficult and often be ignored, especially in countries with large populations, such as China: the detection rate of EGC in China is only 10%, much lower than that in South Korea (50%) and Japan (70%) (3, 4), the diagnosis rate of EGC has great room for improvement. However, the large number of patients, insufficient diagnostic knowledge and experience of physicians, lack of advanced endoscopic equipment, and shortage of endoscopists have seriously affected the improvement of the diagnostic level of EGC in China. These problems are particularly prominent in primary medical institutions (5).

Some studies have reported a false negative rate of 4.6-25.8% in the detection of gastric cancer by esophagogastroduodenoscopy(EGD) (6–14), 71.4% of gastric cancer patients were initially diagnosed with gastritis, ulcers or “suspicious lesions”, with the majority (73%) of errors made by endoscopists (9), technical factors and subjective cognition have significant influence on the screening of EGC (15). The detection of EGC requires not only well-trained endoscopists but also comprehensive knowledge (16), secondly, it is also necessary for endoscopists to avoid the influence of subjective factors, which limit the detection of EGC (17). Therefore, it is very important to develop a tool that has good detection ability and will not be affected by subjective factors to assist endoscopists in the detection of EGC. In recent years, artificial intelligence (AI) based on deep convolutional neural deep learning (DCNN) has come into being, and DCNN has made remarkable progress in various fields, including medicine. In the field of digestive endoscopy, it has been applied to the detection of colonic polyps (18) and the diagnosis of auxiliary capsule endoscopy (19). Based on the above reasons, we constructed an auxiliary diagnosis system for EGC base on DCNN, and tested the diagnostic efficiency of DCNN, aiming to improve the diagnostic efficiency of EGC.

## Methods

### Training dataset preparation

The DCNN was trained using EGD images obtained from the digestive center of Lanzhou University Second Hospital, total 12000 images were selected from the database from January, 2013 to December 2019, including 3400 images of EGC, 8600 images of benign lesions and normal images. All the lesions included in the study were confirmed by biopsy or surgical pathology and the lesion scope was clear, the patient and lesion characteristics of EGC in training set was shown in **Supplementary Material 1**. Postoperative pathological diagnosis included high-grade intraepithelial neoplasia and carcinoma confined to mucosa or submucosa. The equipment for endoscopic images was standard GIF-GIF-260/H290Z, Olympus Medical Systems, Co., Ltd., Tokyo, Japan) and a standard endoscopic video system (EVIS LUCERA ELITE CV-290/CLV-290SL; Olympus Medical Systems), and all images were white light endoscopes without magnification. Images containing poor inflation, halo, blur, defocus or mucus, and post-biopsy bleeding were excluded from the training dataset.

### DCNN training

According to the outcome of pathology, the EGD images were labeled as EGC and other benign lesions, computer engineers will be annotated images for unified clipping, color space transformation, denoising, image morphology operations and normalization of a series of processing, eliminate human and environmental interference, better display image features, enhance the robustness of the algorithm. Algorithm engineers used the DCNN module to test multiple computer models such as DLA34 and Swim Transformer Tiny, and put the training set into the model for training. Through observation and comparison, the 18-layer convolutional neural network model with the optimal accuracy and speed was determined. Input resolution: 512 × 512, batch-size: 32, initial learning rate: 1.25e-4, optimization: Adam 

### DCNN testing

We used standard EGC images independent of the training set to verify the accuracy of DCNN (From January 2020 to October 2020). Our test set was divided into internal test set (ITS)and external test set (ETS). The images of the ETS were from Wuwei Cancer Hospital and Minxian People’s Hospital. The test set excluded postoperative gastric images, magnification, staining endoscopy, mucus and halo images. The patient and lesion characteristics of EGC in ITS and ETS set was shown in **Table 1**. The ITS contains 1289 images (604 EGC) and the ETS contains 542 images (240 EGC). Non-cancer images include ulcers, polyps and chronic gastritis. We identified each lesion area by comparing endoscopic images with the extent of the lesion in the excised specimen, and manually annotated all gastric cancer lesions in the test data set by two experienced endoscopists (L.W and P.W) using a true red rectangular border.

**Table 1:** Patient and lesion characteristics of EGC in Internal test set and External test set.

Patient characteristics	Internal test set (n=134)	External test set (n=48)
Age (years), Mean (range)	60 (27–79)	65 (35-84)
Sex, Number (%)		
Male	92 (68.66)	38 (79.17)
Female	42 (31.34)	10 (20.83)
Size of lesion (mm), Median (range)	15 (4-48.5)	17.4 (6-40.2)
Tumor location, Number (%)		
Cardia_s_ Fundus of the stomach,	18 (13.43)	3 (6.25)
Number (%)		
Body	58 (43.28)	13 (27.08)
Angle	28 (20.90)	12 (25)
Antrum	30 (22.39)	20 (41.67)
Macroscopic type, Number (%)		
0-1	8 (5.97)	1 (2.08)
O-IIa	33 (24.63)	9 (18.75)
O-IIb	7 (5.22)	2 (4.17)
O-IIc	9 (6.72)	0 (0)
0-IIa+O-IIc	47 (35.07)	22 (45.83)
0-Hc+O-IIa	18 (13.43)	10 (20.83)
0-IIb+O-Hc	12 (8.96)	3 (6.25)
O-III	0 (0)	1 (2%)
Differentiation status, Number (%)		
Differentiated	124 (92.54)	41 (85.42)
Undifferentiated	2 (1.49)	1 (2.08)
Mixed	8 (5.97)	6 (12.5)
Depth of tumor, Number (%)		
Tla	128 (95.52)	44 (91.67)
Tlb	6 (8.96)	4 (8.33)

Tla, mucosa; Tlb, submucosa.

### Comparison between the performance of DCNN and endoscopists

Eight endoscopists were selected from two hospitals and divided into the primary group and the expert group. Junior endoscopists (JE) with 2 years of operation experience and less than 1000 cases of EGD operation experience, respectively. Senior endoscopists(SE) have more than 10 years of endoscopic diagnosis and treatment experience, and each of them independently completed at least 80 cases of EGC ESD treatment; the images of the test set are arranged in a random order. Endoscopists individually read the images from the test set and recorded the time required to read the images. At the same time, DCNN recognizes the test set images and records the results.

### Outcome measures and data statistics

**Figure 1:**
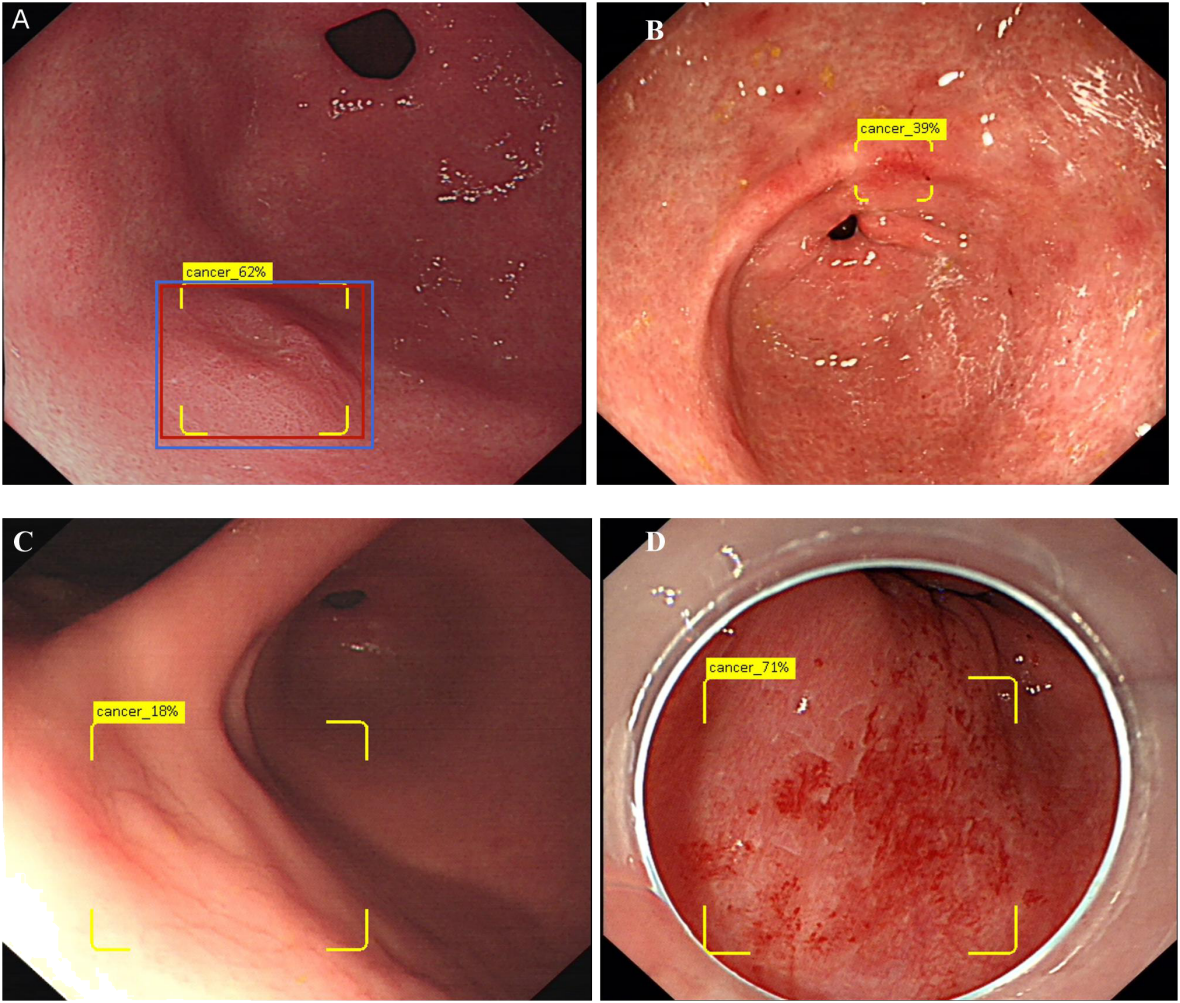
Show the concept of correctly identifies and diagram of DCNN producing false positives. **(A)** Show 0-IIa lesion in the anterior wall of gastric antrum, the red rectangle is the correct marking, the yellow box is the DCNN marking, and the blue rectangle is the endoscopist’s marking. Only when the overlap reaches 50% or more, the diagnosis is correct. The yellow logo shows “Cancer 62%”, indicating that DCNN predicts that the probability of EGC for this lesion is 62%. **(B)** Show an image of falsely diagnosing inflammation as EGC. **(C)** Show an image of DCNN diagnosed the normal mucosa in the reflective area as EGC. **(D)** Show an image of DCNN diagnosed the bleeding mucosa in the reflective area as EGC.

The DCNN showed a 0–100% continuous variable number, which represented a probability score for gastric cancer in each image. Definition of correct answer, for EGC: the correct marking is the red rectangle (according to the results of the ESD postoperative pathology), the yellow rectangle is the DCNN marking and the blue rectangle is the endoscopists marking. When the yellow and red marking overlap is more than 50%, or blue and red marking overlap is greater than 50% is correct(**Figure 1A**); Non-cancerous lesions: The yellow rectangle box is not displayed and the word “cancer” is not displayed.95% confidence intervals (95% CI) using the modified Wald method: Agresti and Coull (The American Statistician. 52:119-126, 1998) Two-tailed unpaired Student’s T-test (chi-square test) was used, with a significance level of 0.05. The accuracy, sensitivity, specificity, positive and negative predictive values (PPV and NPV, respectively) were compared. Interobserver used Cohen’s Kappa coefficient (Kappa value) to assess intra-observer consistency for endoscopists. SPSS 26 (IBM, Chicago, IL, USA) was used to complete all calculations.

### Ethics

The study was approved by the Ethics Committee of the Lanzhou University Second Hospital (No.2022A-004).

## Results

### Characteristics of patients and lesions in the test data set

The characteristics of patients and lesions in the test data set are summarized in **Table 1**, 95.52%patients were mucosal cancer (T1a), 4.48% patients were submucosal cancer(T1b) in ITS, and 91.67%patients were mucosal cancer (T1a), 8.33% patients were submucosal cancer(T1b) in ETS. In terms of histopathological types, 124 (92.54%) patients were differentiated gastric cancer, 10(7.46%) patients were undifferentiated gastric cancer in ITS. Differentiated cancers accounted for 41 (85.42%), and undifferentiated and mixed cancers accounted for 7 (14.58%). The cancer diameter ranged from 4mm to 48.5mm, with a median size of 15mm in ITS, and 6-40.2mm in ETS. The most common Macroscopic type was 0-IIa+IIc, accounting for 35.07% in ITS and 45.83% in ETS, respectively.

### Performance of DCNN model and endoscopists for ITS and ETS

#### DCNN performance

The performances of DCNN model and endoscopist are summarized in **Table 2**. The sensitivity, specificity, accuracy, PPV and NPV of DCNN model in ITS was 88.08%,95% confidence interval (95% CI), (85.24%-90.44%); 89.05%(95%CI, 86.48%-91.19%), 88.60%(95%CI,86.74%-90.22%), 87.64%(95%CI,84.78%-90.04%),89.44%(95%CI,86.90%-91.54%),respectively.And 92.08% (95% CI,87.91%- 94.94%),92.05%(95%CI,88.40%-94.65%), 92.07%(95%CI,89.46%-94.08%), 90.2% (95%CI, 85.79%-93.38%), 93.60(95%CI,90.17%-95.92%) in ETS. The performance of DCNN model in ETS is obviously higher than that in ITS. The average time for DCNN model analysis of each image in ITS and ETS was 0.028s.

**Table 2:** The performances of DCNN and endoscopist in internal test set and external test set.

Competitors	Sensitivity, %	Specificity, %	Accuracy, %	PPV, %	NPV, %	DT(s)
	(95%CI)	(95%CI)	(95%CI)	(95%CI)	(95% Cl)	
**Internal tes**t **set**						
DCNN	88.08	89.05	88.60	87.64	89.44	0.028s
	(85.24-90.44)	(86.48-91.19)	(86.74-90.22)	(84.78-90.04)	(86.90-91.54)	
JE group	69.54^a^	85.69	78.12^s^	81.08	76.13	8.05±0.21^a^
	(66.88-72.07)	(83.74-87.45)	(75.78-80.30)	(78.58-83.35)	(73.94-78.20)	
SE group	89.57^a^	90.00	89,73^a^	88.76	90.73	7.69±0.25^a^
	**(87.71-91.17)**	(88.29-91.48)	(86.79-92.08)	(86.86-90.42)	(89.07-92.16)	
External test set						
DCNN model	92.08	92.05	92.07	90.2	93.60	0.028s
	87.91-94.94	88.40-94.65	89.46-94.08	85.79-93.38	90.17-95.92	
JE group	70.42^a^	82.95	77.40^a^	76.64	77.92	7.98±0.19^a^
	66.18-74.33	79.73-85.74	74.81-79.79	72.47-80.36	74.54-80.96	
SE group	90^b^	90.23	90.13^c^	87.98	91.91	7.50±0.23^a^
	86.97-92.39	87.59-92.36	88.20-91.77	84.79-90.58	89.41-93.86	

a: P<0.01; b: P=0.02; c: P > 0.05. JE(juniorendoscopists); SE(seniorendoscopists); PPV (positive predictive value); NPV(negative predictive value); DT(diagnostic time).

#### Endoscopists performance

The endoscopist’s diagnostic performances are summarized in **Table 2**. The diagnostic time of each image was 8.05 ± 0.21s and 7.69 ± 0.25s for the JE group and SE group in ITS, and there was no significant difference in the diagnostic time between the two, respectively. In ITS,the sensitivity, specificity, accuracy, PPV and NPV of JE group were as follows: 69.54%(95%CI,66.88%-72.07%) 85.69%(95%CI, 83.74%-87.45%) 78.12%(95%CI, 75.78%-80.30%),81.08%(95%CI, 78.58%-83.35%)76.13%(95%CI, 73.94%-78.20%); SE group has a better performance, 89.57(95%CI, 87.71%-91.17%) of sensitivity,90.00%(95%CI, 88.29%-91.48%)of specificity,89.73% (95%CI,86.79%-92.08%) for accuracy,88.76% (95%CI, 86.86%-90.42%)for PPV and 90.73%(95%CI, 89.07%-92.16%) for NPV. The sensitivity and accuracy of the SE group were significantly higher than those of the JE group(P<0.01), The sensitivity of SE group was higher (by20.03%95%CI, 17.03%-23.42%,P<0.01) than JE group, and the accuracy of SE group was higher (by11.61%,95%CI, 10%-13.51%,P<0.01) than JE group. In the ETS, the diagnostic time for each image in the JE and SE groups was 7.98 ± 0.19s and 7.50 ± 0.23s, respectively, and there was no significant difference in diagnostic time between the two group, the performance of SE group was significantly higher than that of JE group, the sensitivity of SE group was higher (by19.58%,95%CI, 15.04%-25.09%,P<0.01) than that of JE group, and the accuracy of SE group was higher (by12.73%,95%CI, 10.17%-15.81%, P<0.01) than that of JE group. In terms of the ITS and the ETS, the diagnostic efficacy of endoscopists in the ETS was higher than that in the ITS.

In terms of diagnostic consistency, in the ITS, the DCNN model and endoscopist’s pairwise Kappa values ranged from 0.765 to 0.913, while the endoscopist’s diagnostic Kappa values ranged from 0.735 to 0.959. The diagnostic consistency was reasonable. The mean value of Kappa between DCNN model and endoscopist was 0.8794, JE-1 was 0.8424, JE-2 was 0.871, SE-1 was 0.8868, and SE-2 was 0.878. In the ETS, the Kappa values of DCNN model and endoscopists ranged from 0.676 to 0.981, while the diagnostic Kappa values of endoscopist ranged from 0.682 to 0.950. The mean value of Kappa between DCNN model and endoscopist’s was 0.8638, JE-3 was 0.8106, JE-4 was 0.8418, SE-3 was 0.8832, and SE-4 was 0.8686.

#### Comparison of DCNN and endoscopist performance

**Figure 2:**
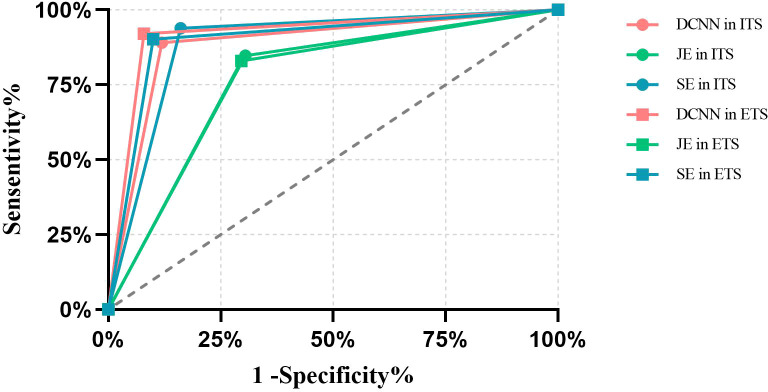
ROC curve of DCNN model and endoscopists in internal test sets and external test sets.

The receiver operating characteristic (ROC) curve of DCNN model and endoscopist’s diagnostic effectiveness is shown in **Figure 2**. In ITS, the area under ROC curve (AUC) of the DCNN model, JE group and SE group were 0.8857 (95%CI,0.8655-0.9058),0.7710 (95%CI,0.7443-0.7978) and 0.8890(95%CI,0.8690-0.9091),respectively. In ETS, AUC of the DCNN model, JE group and SE group were 0.9207 (95%CI,0.9020-0.9394), 0.7668(95%CI,0.7372-0.7964) and 0.9012 (95%CI,0.8805-0.9218), respectively. DCNN model was significantly faster than all endoscopists in test sets. The sensitivity of the DCNN model was 18.54% (95%CI, 15.64%-21.84%,P<0.01) higher than that of the JE group and 0.33%(95%CI, 0.06%-1.05%, P>0.05) lower than that of the SE group in the ITS. In the ETS, it was 21.67% (95%CI, 16.90%-27.32%, P<0.01) higher than that in the JE group and 2.08%(95%CI, 0.75%-4.92%, P>0.05) higher than that in the SE group. In terms of accuracy, DCNN model was 10.47%(95%CI, 8.91-12.27%,P<0.05) higher than that of JE group, and 1.16% (95%CI, 0.69%-1.93%,P > 0.05) lower than that of SE group in the ITS; In the ETS, it was 14.58%(95%CI, 11.84%-17.81%, P<0.01) higher than JE group and 1.94% (95%CI,1.25%-2.96%,P > 0.05)higher than SE group.

### Cause of false positives and false negatives

In order to further analyze the causes of false positives and false negatives produced by DCNN model and endoscopists, we summarized it in **Tables 3**, **4**. The first cause for false positive of DCNN model is Gastritis (redness, atrophy, intestinal metaplasia)(44% and 62.5%,respectively),which were also the most reasons for endoscopists(59.29% and 68.52%).Mucus (10.67%) was the secondary cause of in the ITS, while ulcer (12.5%) was the secondary cause in the ETS, which we found that the surface appearance of ulcers were very similar to that of gastric cancer. The third false-positive factor was folding and foam (9.33% in the ITS) and blood (8.33% in the ETS). For endoscopists, ulcer was the second reason (11.43% and 18.52%). However, compared with the DCNN model, endoscopists rarely mistake mucus, foam, and folding for EGC.

**Table 3:** Details of DCNN model and false positive images of endoscopists.

	Internal test set		External test set	
Cause	DCNN, n (%)	Endoscopists, Total n	DCNN,n	Endoscopists, Total n
		(%)	**(%)**	(%)
Total number	75	280	24	162
Gastritis (redness, atrophy, intestinal metaplasia)	33 (44)	166 (59.29)	15 (62.5)	111 (68.52)
Mucus	8 (10.67)	2 (0.71)	1 (4.17)	4 (2.47)
Fold	7 (9.33)	11 (3.93)	0 (0)	7 (4.32)
Foam	7 (9.33)	4 (1.43)	1 (4.17)	0 (0)
Halation	5 (6.67)	5 (1.79)	0 (0)	0 (0)
Blood	4 (5.33)	8 (2.86)	2 (8.33)	4 (2.47)
blood vessel	4 (5.33)	0 (0)	0 (0)	0 (0)
Ulcer	3 (4)	32 (11.43)	3 (12.5)	30 (18.52)
Xanthoma	2 (2.67)	11(3.93)	1 (4.17)	3 (1.85)
Normal anatomical structure (cardia, pylorus, angulus)	1 (1.33)	0 (0)	0 (0)	0 (0)
Hyperplastic polyp	1 (1.33)	30(10.71)	0 (0)	2 (1.23)
Scar	0 (0)	11(3.93)	1 (4.17)	1 (0.62)

**Table 4:** Details of false negative images by DCNN model and endoscopists.

	Internal test set		External test set	
Cause	DCNN, n (%)	Endoscopists, total n (%)	DCNN, n (%)	Endoscopists, totaln (%)
Total number	72	562	19	190
Small (< 10mm)	28 (38.88)	112 (19.93)	4 (21.05)	16 (8.42)
visual angle	18 (25)	50 (8.90)	1 (5.3)	2 (1.05)
Distant	12 (16.67)	90 (16.01)	3 (15.79)	12 (6.32)
Tangential Line	5 (6.94)	12 (2.14)	2 (10.53)	4 (2.1)
Ulcer	4 (5.56)	91 (16.19)	3 (15.79)	28 (14.74)
Adenoma-like	2 (2.78)	16 (2.85)	1 (5.3)	4 (2.1)
Inflammation-like	2 (2.78)	184 (32.74)	2 (10.53)	108 (56.84)
Blood	1 (1.39)	2 (0.36)	1 (5.3)	6 (3.16)
Scar-like	0 (0)	5 (0.89)	0 (0)	5 (2.63)
Others	0 (0)	0 (0)	2 (10.53)	5 (2.63)

**Figure 3:**
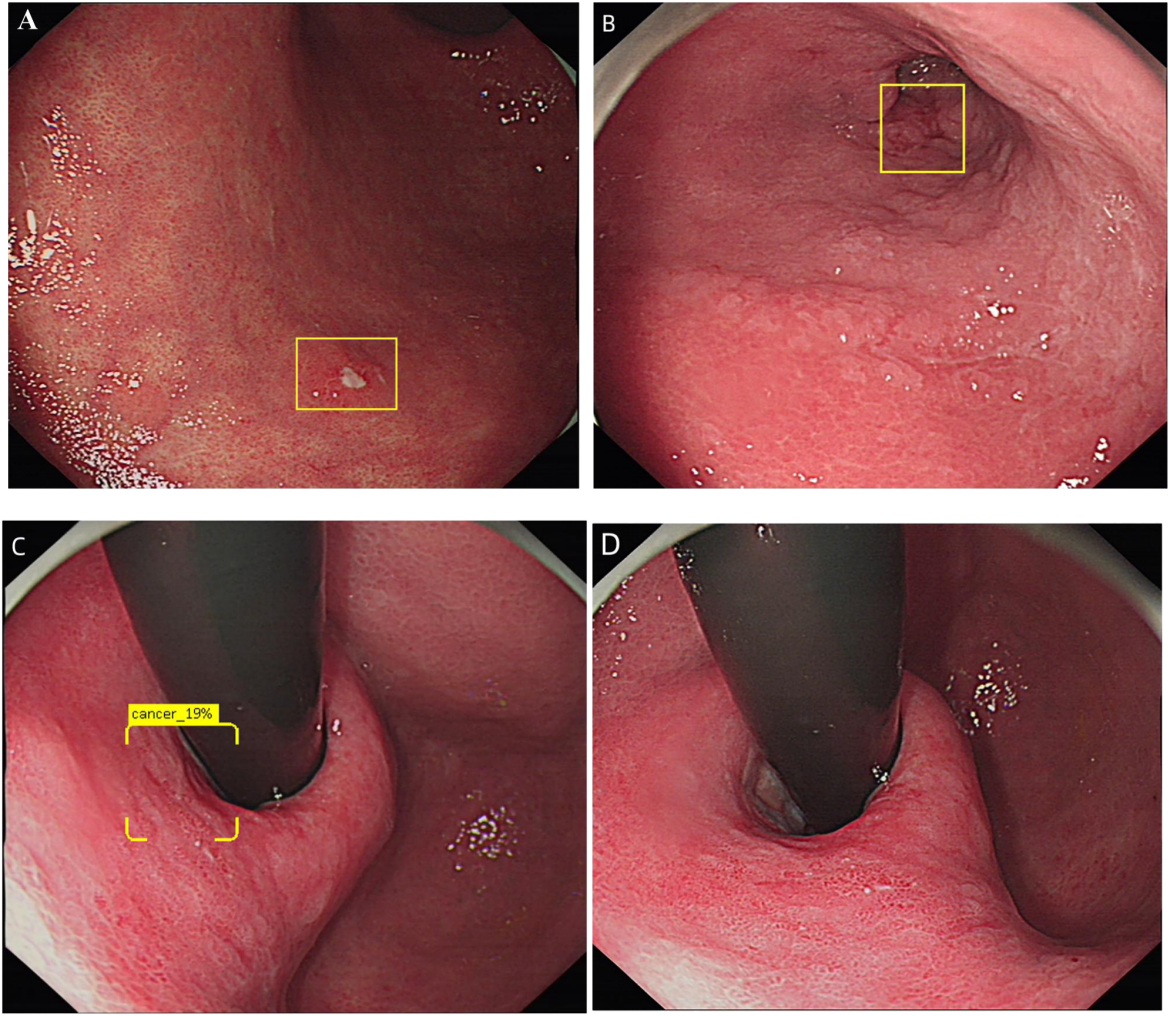
Diagram of DCNN producing false negatives. **(A)** Show an image that the lesions were too small to be recognized by DCNN. **(B)** Show an image of the lesions were too far to be recognized by DCNN. **(C, D)** Shows images taken from different shooting angles of the same lesion. **(C)** was effectively recognized as EGC by DCNN, while **(D)** was not recognized by DCNN.

**Table 4** summarizes the causes of false negatives, the first reason for the false negative in DCNN model was that the diameter of the lesions was less than 10mm (38.88% and 21.05%, respectively). And the 32 images were from 14 patients respectively, DCNN model could identify some of the images in these cases, however the long shooting distance combined with the small diameter of the lesions was the biggest reason for the error recognition of DCNN model. The second factor was visual angle (25% in the ITS), distance and ulcers (15.79% in the ETS, respectively). Different shooting angles resulted in incomplete identification of multiple images of the same lesion, some lesions in these unrecognized images were relatively flat (type 0-IIb), some of the light increased as the shooting angle changed. The third factor of false negative was distant (16.67% in ITS), tangential line and inflammation-like(10.53%, respectively). For endoscopists, the biggest factor of false negative is inflammation-like (32.74% and 56.84%). Examples of false positive and false negative by DCNN model was shown in **Figures 1B–D**, **3A–D**.

## Discussion

In the world, only Japan and South Korea have relatively high diagnosis rate of EGC, while European and American countries have not carried out large-scale endoscopic screening of EGC. China has a large population, and the incidence of gastric cancer accounts for 1/4 of the world, but the diagnosis rate of EGC is only 10% (3, 4), it is far behind Japan and South Korea. The difficulty in treatment of EGC lies in early detection. EGD is the only effective tool to identify EGC. Although standardized training of EGD can improve the diagnosis rate, However, the time of training curve is long, and the scope of standardized training is limited (20, 21), in addition, there is a serious shortage of specialized endoscopists in China, the overall level of diagnosis rate in EGC is low, and it is difficult to improve the diagnosis rate of EGC in a short period of time. Therefore, how to quickly shorten endoscopists training time and improve the level of diagnosis rate of EGC is an urgently problem for us to solve.

Although various image enhancement techniques have been developed and applied, white light imaging(WLI) is the first step in standard EGD (16), The use of image enhancement technology is considered only after suspicious lesion under WLI. It has been reported that the sensitivity of WLI for EGC is 33%-75% (22), On the other hand, diagnosis depends on the experience and subjective awareness of the EGD operator (17). Therefore, through the training of images, we developed the DCNN system to assist the diagnosis of EGC in WLI. The sensitivity of DCNN model is 88.08% and 92.08%, which was significantly higher than that of all endoscopists, and its recognition time was significantly shorter than that of all endoscopists.

We found that ulcers (11.43% and 18.52%) were an important cause of false positives produced by endoscopists, as well as a cause of missed diagnosis (16.19% and 14.74%), which means that endoscopists have difficulty in distinguishing ulcers from EGC. Ulcers were included as negative controls in our DCNN model training. Therefore, compared with endoscopists, the proportion of false positives in DCNN model due to ulcers was lower than that of endoscopists (4%-12.5% vs.11.43%-18.52%). This means that our DCNN model can help endoscopists reduce the incidence of such errors. Of course, our DCNN model also has a certain false negative rate. Among them, 38.88% and 21.05% are lesions smaller than 10mm. Considering that it is difficult for even experienced endoscopists to diagnose small lesions, the development time of intratumoral cancer is 2-3 years, we speculate that this limitation can be confirmed by annual upper gastrointestinal endoscopy and biopsy (23), and for the update iteration of DCNN, we will also increase the training of small lesions. 25% and 5.3% false negatives are difficult to identify because of different angles of view. However, DCNN can identify at least two images of a patient from different angles, therefore, we speculate that the retention of suspicious lesions from multiple locations and angles can make up for this defect of DCNN. The lesion distance is the third major reason leading to false negative of DCNN. Although the morphology of these lesions is prominent, but the images were taken as a far distance, and the color, texture and other features of the lesions were not obvious, therefore it is easy to be ignored by the DCNN. Another rare reason for false negatives in DCNN is that lesions are similar to inflammatory changes, but they are the most common cause for endoscopists. This finding suggests that endoscopists may misdiagnose inflammation, but DCNN does not miss lesions (2.78% vs. 32.74% in ITS, 10.53% vs.56.84% in ETS).

The most common causes of false positives are mucosal redness, atrophy, and intestinal degeneration. Even experienced endoscopists can hardly distinguish these lesions by a single WLI without magnifying endoscopy. 29.33% and 8.34% of false positives are due to mucus, foam and folding. DCNN is more likely to be affected by the above factors, while endoscopists are less affected by these factors, which means that DCNN may be more affected by the background in the stomach in the actual application process. However, as demonstrated by Mori et al. (24) these limitations can be reduced by mucosal irrigation, the use of antifoaming agents and adequate gas injection, as well as the application of a large number of images for training in the real process of EGD.

For specificity and PPV, Although SE performed better on specificity and PPV in ITS, the DCNN model was higher than that of JE, our image of training set goes through carefully selected, excluded those poor quality of the image images, containing mucus, bubble, folding and dizzy light images, We believe that if we strengthen the training of false positive images, these problems will be solved (25). DCNN is more sensitive and can identify more EGCs than experienced endoscopists, especially for JE. In addition, the sensitivity and PPV of expert endoscopists are significantly higher than those of JE, so DCNN may be more helpful to those endoscopists with limited experience.

We reviewed the relevant literature and found that some AI have a sensitivity of up to 90% (26–28). However, the control groups in these studies only included normal or chronic gastritis, not ulcerative lesions that endoscopists are more likely to confuse. and there studies focused on the sensitivity of detecting gastric cancer as a whole, including advanced gastric cancer, and were not compared with endoscopists. Moreover, most studies did not analyze the causes of false positive and false negative for endoscopists and DCNN, so as to carry out targeted strengthening training. The sensitivity found in this study appears to be lower in ITS than those studies for the following reasons: first, only EGC images were included in this study, and intermediate and advanced gastric cancer were not included. Second, sensitivity was calculated per lesion, not per image in those studies, that is, if at least one image of gastric cancer is recognized in multiple images of the same lesion, the diagnosis is considered correct. Third, in these studies, the concept of correct diagnosis is that if the image area identified by DCNN overlaps slightly with that of EGC, it is considered correct. However, in our study, only the image range identified by DCNN overlapped by more than 50% with the image range marked by endoscopist was considered correct, while occasionally marked or not marked enough, we considered it incorrect. Recently, A multicenter study reported (29) that DCNN assisted diagnosis of upper gastrointestinal tumors, including gastric cancer shows diagnostic accuracy of DCNN was up to 90%, and the sensitivity was comparable with that of expert endoscopists. However, in this study, the rate of advanced gastric cancer was relatively higher, while the early gastric cancer was only 18.6%. Our research aims to find more EGC that endoscopists are prone to misdiagnose.

In terms of stability evaluation, SE group with higher diagnostic accuracy has better diagnostic consistency than JE group. According to the general guidelines of ICC standard (30), there are considerable differences in the diagnosis consistency among endoscopists, which is not clearly related to professional knowledge and experience. Due to the subjective interpretation of the characteristics of EGC and the different learning curve in the diagnosis of EGC, objective diagnosis is very necessary (9). The DCNN system achieves perfect observer protocol (Kappa 1.0) without interference of subjective judgment. The EGC detection system based on the DCNN has sufficient and consistent diagnostic performance, eliminating some diagnostic subjectivity. Moreover, the DCNN system is very helpful for JE, this is consistent with the research of Ikenoyama et al. (31) DCNN may be a powerful tool to assist endoscopists, especially JE in detecting EGC. The shorter screening time and fatigue free DCNN may enable rapid surveillance of EGC. More importantly, the diagnosis of EGC by DCNN can be fully automated and online, which may facilitate the development of telemedicine and thereby alleviate the problem of a shortage of endoscopists.

Yet, the study has several limitations, first of all, our DCNN only trained WLI images, it can provides the first step of EGC detection, which is also the most important step, but it did not trained narrow-band images (NBI) and the magnifying endoscopy (ME). However. in past research report, endoscopic image enhancement is rarely used, unless there is a suspicious lesion found in the WLI (32). In addition, a multicenter study showed no significant difference in the diagnostic efficacy of nonamplified NBI and WLI in EGC (33). Moreover, in reality, unless use the NBI routinely in esophageal observation, It is generally not used in endoscopic examination unless suspicious lesions are found under WLI (32). Secondly, we only use Olympus 260 or 290 series gastroscope system, without other brands of endoscopes such as Fuji Endoscope, this may reduce the efficiency of DCNN. Third, in the control group of gastric cancer detection dataset, we eliminated most of the images containing mucus and halo, and the diagnostic sensitivity of DCNN may be lower in the real world. Fourth, static images are used in the training and testing sets of this study, and video images can improve the performance and present the real scene, we plan to use video as a validation set in the future, which will be used as another separate study. Fifth, in this study, DCNN model missed diagnosis of small lesions, increasing the number of training of small lesions and flat lesions, as well as the number of negative control images will help improve the diagnostic efficiency of the model.

In conclusion, we constructed an assist EGC detection system based on DCNN and compared the diagnostic ability of DCNN and endoscopists. It has excellent diagnostic sensitivity, fast diagnostic characteristics, achieved a perfect observer protocol. It can help endoscopists (especially JE) to find more EGC. We believe that DCNN will contribute to the overall improvement of the diagnosis rate of EGC, and serve as an assisting work to help improve the diagnosis rate of EGC.

## Data availability statement

The original contributions presented in the study are included in the article/**Supplementary Material**. Further inquiries can be directed to the corresponding author.

## Ethics statement

Written informed consent was obtained from the individual(s) for the publication of any potentially identifiable images or data included in this article.

## Author contributions

JF designed the experiment and drafted this article. SY and YZ were responsible for the collection of training set images. LQ was responsible for the submission of ethical approval documents. YF nd YY were responsible for the annotation of the images. LG, XL, WL and PW were responsible for the recognition of the test set images. YB and LZ were responsible for the algorithm training of DCNN. XH was responsible for the project coordination. All authors contributed to the article and approved the submitted version.
