# Supplementary material for: A system based on deep convolutional neural network improves the detection of early gastric cancer
Source: Front Oncol. 2022 Dec 22;12:1021625. doi: 10.3389/fonc.2022.1021625 (PMC9815521; doi:10.3389/fonc.2022.1021625)
Supplement: Supplementary file 1 [file Table_1.pdf]

Supplementary Material 1. Patient and lesion characteristics of EGC in Training set.

Patient characteristics	Training set(N=652)
Age(years)， Mean(range)	61(26-88)
Sex， Number(%)	
Male	453(69.48)
Female	199(30.62)
Size of lesion(mm), Median(range)	2.1 （5-40.2）
Tumor location， Number(%)	
Cardia、Fundus of the stomach， Number(%)	98(15.03)
Body	178(27.30)
Angle	163(25)
Antrum	213(32.67)
Macroscopic type， Number(%)	
0-I	28(4.29)
0-IIa	123(18.87)
0-IIb	33(5.06)
0-IIc	41(6.29)
0-IIa+0-IIc	187(28.68)
0-IIc+0-IIa	163(25)
0-IIb+0-IIc	47(9.21)
0-III	30(4.6)
Differentiation status， Number(%)	
Differentiated	512(78.53)
Undifferentiated	78(11.96)
Mixed	62(9.51)
Depth of tumor， Number (%)	
T1a	582(89.26)
T1b	70(10.74)

T1a, mucosa; T1b, submucosa;
